# Self-organization in Slovenian public spending

**DOI:** 10.1098/rsos.221279

**Published:** 2023-08-02

**Authors:** Jelena Joksimović, Matjaž Perc, Zoran Levnajić

**Affiliations:** ^1^ Faculty of Information Sciences in Novo Mesto, Ljubljanska cesta 31A, Novo Mesto 8000, Slovenia; ^2^ Faculty of Natural Sciences and Mathematics, University of Maribor, Koroška cesta 160, Maribor 2000, Slovenia; ^3^ Department of Medical Research, China Medical University Hospital, China Medical University, Taichung 404332, Taiwan; ^4^ Alma Mater Europaea, Slovenska Ulica 17, Maribor 2000, Slovenia; ^5^ Complexity Science Hub Vienna, Josefstädterstrasse 39, Vienna 1080, Austria; ^6^ Department of Physics, Kyung Hee University, 26 Kyungheedae-ro, Dongdaemun-gu, Seoul, Republic of Korea

**Keywords:** corruption, public spending, Slovenia, self-organization, power law, preferential attachment

## Abstract

Private businesses are often entrusted with public contracts, wherein public money is allocated to a private company. This process raises concerns about transparency, even in the most developed democracies. But are there any regularities guiding this process? Do all private companies benefit equally from the state budgets? Here, we tackle these questions focusing on the case of Slovenia, which keeps excellent records of this kind of public spending. We examine a dataset detailing every transfer of public money to the private sector from January 2003 to May 2020. During this time, Slovenia has conducted business with no less than 248 989 private companies. We find that the cumulative distribution of money received per company can be reasonably well explained by a power-law or lognormal fit. We also show evidence for the first-mover advantage, and determine that companies receive new funding in a way that is roughly linear over time. These results indicate that, despite all human factors involved, Slovenian public spending is at least to some extent regulated by emergent self-organizing principles.

## 1. Introduction

How to best spend public money? The question is not as old as time, but it certainly is a strongly contested issue. Do we fix potholes and erect new playgrounds, or do we beef up the police and the military? Do we invest in science and technology, or should we attend to our ageing population? The choices are diverse and many, and so are the opinions and policies that ultimately determine who benefits from the public money [1]. Most delicate in this is the issue of paying private companies and contractors from public funds and the state budget. How should we choose which private business to entrust with, say, building a new school? Decisions to allocate public money to the private sector also raise concerns about transparency, even in the most developed democracies.

In light of this, it seems almost heretical to consider a concept as pure as *self-organization* [2] to be at play in the dynamics of public spending. But the broader history of this natural phenomenon provides some rationale for such a hypothesis [3,4]. In his 1934 work *The Theory of Economic Development*, Schumpeter [5] elaborated on the concept of proportional growth, wherein he argued that the more wealth a business has, the more it is likely to attract it in the future. This work was predated by the Gibrat rule of proportional growth [6], which posits that the proportional rate of growth of a company is independent of its size. Even earlier, in 1922, and motivated by observations of the statistics of biological taxa [7], the Yule process was proposed as possibly the first mechanism behind the power laws in nature [8–12]. It relied on the assumption that an initially small (biological) advantage is likely to snowball over time [13]. In more contemporary research, growth and preferential attachment became synonymous for the Barabási–Albert [14] model, which describes the emergence of scaling and power-laws in real networks. This also became a method of spotting various forms of self-organization not just in natural systems, but also in social systems [11,15–17]. The circle can be closed with the quote attributed to St. Matthew: ‘For to all those who have, more will be given’. It is the saying behind the well-known *Matthew effect*, which describes the general pattern of self-reinforcing inequality related to economic wealth, political power, prestige, knowledge and many other social resources [3].

Here we ask: Are there self-organizing mechanisms in the process of allocating public money to private companies? If so, can they be detected by analysing data on public spending? In what follows, we offer evidence in favour of this hypothesis using a dataset from Slovenia, which keeps excellent records of this kind of public spending.

Apart from looking for self-organization, there is another important reason to examine the dynamics of assigning public money to the private sector: concerns about corruption. According to the World Bank estimates, bribes alone amount to about 1 trillion USD around the world [18,19]. Transparency International claims that corrupt officials in developing countries receive as much as 40 billion USD in bribes per year, and that nearly two out of five business executives pay bribes when dealing with the incumbent public officials [20]. Researchers have documented the effects of corruption in many forms. It limits economic growth [21–25], it decreases returns on public investments [26] and it promotes socioeconomic inequality [23,27]. By studying the statistical patterns in allocation of public funds, we are opening new avenues for designing better methods for detecting suspicious allocations (although we do not expect to actually find evidence of corruption in self-organization mechanisms).

### 1.1. Data

Motivated by these considerations, we set out to test the presence of self-organization in the public spending of the Republic of Slovenia (a note on terminology: we call ‘public spending’ the public money allocated to private companies, whereby we exclude other forms of allocations that are usually considered as public spending [28]). Being a (relatively) small nation, Slovenia keeps excellent records of public allocations to the private sector via the Commission for the Prevention of Corruption of the Republic of Slovenia (CPC) [29,30]. It is an independent agency with a broad mandate to prevent and investigate corruption and other breaches of ethics and integrity, with a special focus on transparency of public spending. The CPC gathers the data from nine different public institutions in Slovenia, including the Ministry of Finance, Public procurement portal and Public Payments Administration. In particular, all private companies registered in Slovenia are under a legal obligation to report the exact information on any business undertaken using public funds. Hence, the CPC keeps track of all transactions where public money is being spent on the private sector.^1^ For transparency motives, all these data are publicly accessible on the CPC website via Erar software.^2^

**Figure 1.**
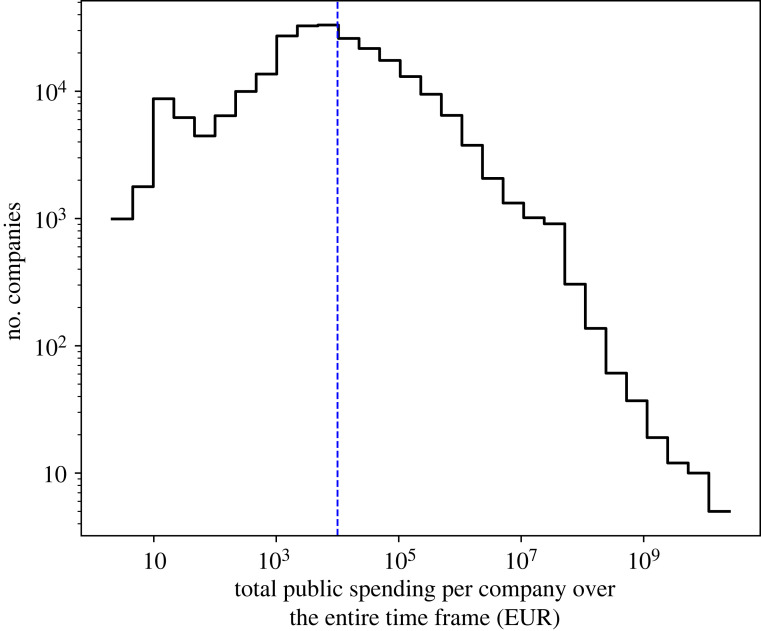
Histogram of total public spending in favour of private companies over all 209 months (log–log scale). Also shown is our cut-off at 10 000 EUR; we excluded companies that in total received less than 10 000 EUR, i.e. left of the vertical line.

Upon signing the required contract, we received our dataset from the CPC itself. Although the companies’ names are public, we requested the dataset to be anonymized for avoidance of bias. The signed contract details how the data can and cannot be used, and deals with all relevant ethical concerns. The received dataset includes all public-to-private transactions from January 2003 to May 2020. During these 209 months, the Republic of Slovenia has conducted business with exactly 248 989 companies. To avoid noise and uncertainties, we excluded from further analysis companies that in this period made less than 10 000 EUR. Above this cut-off in total spending per company translates to 105 086 companies, to which we focus in the analysis that follows. To illustrate this, we show in figure 1 the distribution of companies according to their total revenue. The cut-off is shown as the vertical blue line. Our data can be thought of as a matrix of dimensions 209 × 105 086, i.e. with companies in rows and time (month) in columns. In this matrix, each element is the amount of money (in EUR) that a company received from all public bodies during one of these 209 months. In other words, for any given company, we have a time series with 209 values, each value representing the volume of business conducted using public funds over that month.

This data is possibly unique in the world. Its completeness and precision allows us to carry out this work. As we show in what follows, we found a heavy-tailed distribution of total public spending per company versus company rank that can be fitted reasonably well by a power law. There is evidence for the first-mover advantage [31,32], meaning that companies that started doing business early tend to accrue more. We also find that the attachment rate of new companies is roughly linear. Taken together, these results indicate that in the case of Slovenia, public spending is guided by self-organizing principles, not unlike those hypothesized by Schumpeter [5] and Gibrat [6].

## 2. Results

We organize our findings into three main categories, following the steps presented in figure 7, in §3. This section also includes all other technical details about our methodology. As the figure shows, we ask three questions to determine whether Slovenian public spending exhibits self-organization:

— *Q1: Is there evidence of first-mover advantage?* This phenomenon, in which early adopters of a new technology or practice have an advantage over later adopters, is a key indicator of self-organization [2].
— *Q2: Does the distribution of public spending fit to a power law or lognormal?* Systems that self-organize usually display power laws, which are characterized by a small number of entities with numerous connections and a large number of entities with few connections [11,33]. However, it is important to consider alternative distributions such as lognormal, which may also be indicative of self-organization [34].
— *Q3: Does this system grow via linear preferential attachment?* Is the likelihood of a company attracting new business (at least roughly) proportional to the amount of business it has won so far (at least for the top-ranked companies)? If so, this is another strong indication of self-organization [35].

While there is no one-size-fits-all approach to identifying self-organization, these criteria provide a starting point for investigating the emergence of regularity in complex systems in the absence of external or central guidance [12]. If we can affirmatively answer all three questions, then we can conclude that our public spending data exhibits self-organization. The next three subsections are devoted to these questions. To wrap up our analysis, we conclude the Results section with another short subsection. There, we look at the descriptive statistics regarding the overall behaviour of companies during the analysed period, including rates of entry and exit.

### 2.1. First-mover advantage

This section is devoted to our first question—*Q1*. We want to find if there are signs that entering this system early gives you an advantage over others.

#### 2.1.1. Overall dynamics of public spending

**Figure 2.**
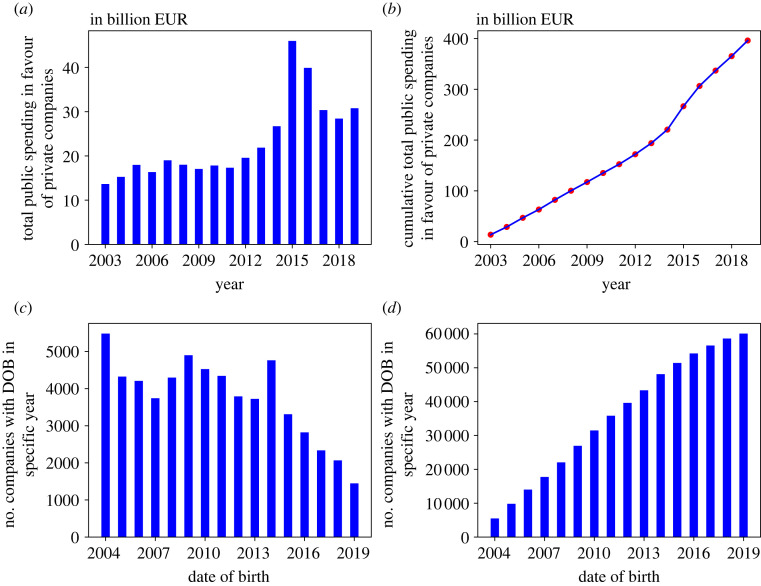
Overall dynamics of public spending. (*a*) Yearly dynamics of public spending in the entire private sector from 2003 to 2019. (*b*) Cumulative spending over the same period. (*c*) Cumulative total number of companies with date of birth (DOB) in specific year. (*d*) Year-by-year number of companies with specific DOB.

We begin by examining the overall behaviour of public spending during the given time interval. For each year, we sum the amount of money received by all companies that did business with the state. That is to say, for each year we obtain the total funds that the private sector jointly received (we exclude the year 2020 since the data are available only until May). The result is shown in figure 2*a*. The total spending mainly stagnated or slowly increased from 2003 to 2015. It then increased significantly (almost doubled), and then started to decrease from 2016 on, but still remained at a level higher than before 2015. Finally, there is a small increase in 2019 compared to 2018. It appears that the economic crisis of 2008 [36] has not significantly influenced these dynamics, but it might have slowed it down and/or somewhat delayed it. Overall, it is clear that since 2003, the Slovenian state is doing more and more business with the private sector. This is also visible in figure 2*b*, which shows the cumulative spending: the funds received cumulatively until (and including) each of the years. The curve displays a relatively smooth upward trend, roughly linear, with a somewhat faster slope from 2015 on.

This begs the question: Does public spending always go to the same companies or do newcomers on the stage get a fair chance? To answer this, we check how the above dynamics are reflected in the number of new companies that are being established each year. To set our terminology, for any company, we call ‘date of birth’ (DOB) the year in which that company had its first transaction with the state and it first appeared in our dataset [37]. We compute the DOBs for all companies and show the result in figure 2*c* (yearly) and figure 2*d* (cumulatively). Figure 2*d* shows that the number of companies doing business via public budgets has steadily grown since 2004, although the rate is somewhat slowing down. Figure 2*c* gives us an even clearer look, showing the exact number of ‘newborn’ companies year by year. Most of the companies were established in 2004; more than 5000 of them. Since then, this trend fluctuates but overall slows down. Exceptions are 2009 and 2014, when we have local peaks of more companies being born than in previous years. It seems that companies with a DOB in 2004 have not immediately received the biggest portion of their revenue. However, being established so early places them in a good position to make larger deals than their competitors with later DOBs. This suggests that DOB does play a role in a company’s propensity to do business with the state.

To test this further, we examine how the annual (yearly) revenue depend on companies’ DOBs: Is it true that older companies (typically) make larger annual amounts than younger companies? This is to say, we put together all companies with the same DOB and study the distribution of their annual revenue (across both companies and years). Since our dataset starts with 2003, all the companies with DOBs before 2003 appear in our data as if their DOB was 2003. In other words, for a company with a DOB in 2003, we have no way of telling whether the DOB is truly 2003 or before that. For this reason, we start from 2004 in this analysis.

**Figure 3.**
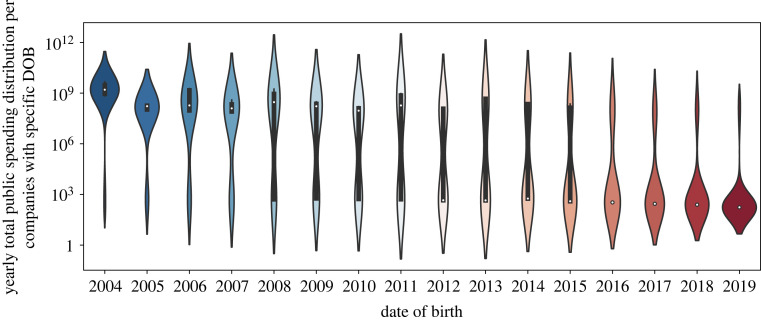
Violin plot of annual (yearly) public spending in favour of private companies depending on their DOBs. Each violin illustrates the distribution of annual revenue in favour of companies with a DOB in that year. So, the thickest part of a violin shows the typical annual amounts received by those companies (note the log scale on the *y*-axis). For example, the companies with a DOB in 2004 usually received between 10^8^ EUR and 10^10^ EUR per year, whereas companies with a DOB in 2019 received only about 100 EUR per year.

Results are shown in figure 3, where the distribution of annual revenue is shown as a vertical ‘violin’—the horizontal thickness of a violin captures the distribution for all companies with that DOB.^3^ Companies with a DOB between 2004 and 2007 earn significantly larger annual amounts than companies with later DOBs. Companies with a DOB in 2008—2014 earn some large and some small annual amounts, while companies with a DOB in 2015—2019 earn almost exclusively small annual amounts. Also interesting is that even though public payments are biggest in 2015 and 2016 (cf. figure 2*a*), amounting to almost 90 billion EUR, this boost in public spending seems to have largely gone to older companies rather than newly joined ones. Moreover, these ‘violin bellies’ gradually get bigger for companies with later DOBs. This means that newer companies tend to all receive similar total amounts, whereas older companies have a wider distribution of total amounts (additional evidence for this can be found in the figure included in the electronic supplementary material).

This result strongly suggests the presence of a first-mover advantage: the total revenue of companies correlates with the time when they joined the system. This indicates that being an early entrant confers an advantage. It is also interesting to check how this depends on the actual amounts. Are companies that started with larger amounts the sole beneficiaries of significant growth in the future, while those that started with smaller amounts remain stagnant? Alternatively, do smaller companies have the potential to change their situation and achieve growth? We address this inquiry next by analysing the growth rates of the highest-ranked companies.

#### 2.1.2. Growth rates of most successful companies

**Figure 4.**
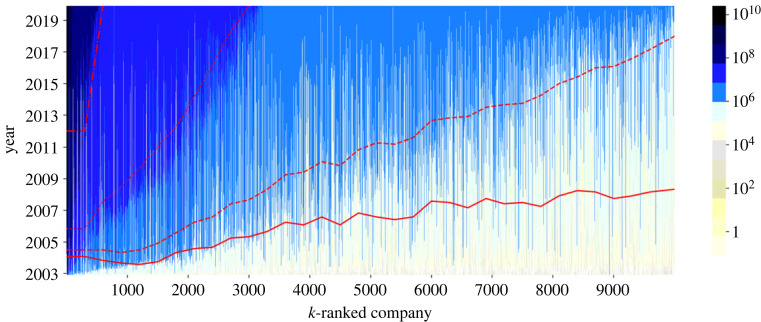
Colour map of growth of public spending per individual companies. Ten thousand companies are ranked according to their total revenue, from *k* = 1 (most successful) to *k* = 10 000 (least successful). They are arranged in this order from left to right, whereby each (thin) vertical line refers to one company. The colour bar shows the spending (log scale), which increases in favour of all companies over time, flowing from the bottom to the top. Red lines represent the thresholds when companies cross (from the bottom to the top) 100k EUR, 1M EUR, 10M EUR and 100M EUR in terms of cumulative spending. These calculations are performed via non-overlapping windows, each containing 300 consecutively ranked companies.

We now look at the growth rates of individual companies over the examined time frame. For better focus, we consider only the 10 000 most successful companies in terms of total revenue. We rank them from most to least successful (*k* denotes the rank). We show this via the colour map in figure 4, whereby each vertical line refers to a single company. Ten thousand parallel lines represent 10 000 companies. Lines are squeezed next to each from left (most successful, rank *k* = 1) to right (least successful, rank *k* = 10 000). Gradual darkening of each line illustrates the accumulation of income by that company (see colour bar). Naturally, vertical lines start with the company’s DOB (and are zero before the DOB) and gradually become darker as the company conducts more business with the state. There is a robust pattern: companies with higher ranks and total revenue in hundreds of millions Euros tend to start accumulating much earlier. By contrast, companies with lower ranks tend to start doing business later, meaning that they ultimately accumulate smaller amounts. There are exceptions to both, but not very many overall. This ‘orderliness’ again suggests that self-organizing mechanisms are at play.

The full red line in figure 4 represents the average time when companies cross the 100k EUR threshold in terms of total revenue. Apart from some noise, this line displays (almost) linear growth with rank, which is much smoother than expected in general. This means that the time to reach 100k EUR grows (roughly) linearly with the rank. The story is the same with the next (dashed) red line, which refers to the 1M EUR threshold, except that the growth is steeper. This means that for companies with higher ranks, it takes less time to go from 100k to 1M EUR. This trend is present for the next red line marking the 10M EUR threshold, etc. As growth and cumulative advantage are the two crucial factors for the emergence of scaling in random networks [38–40], our current findings move us to the next question—*Q2* in which we check if our data follows the power-law distribution.

### 2.2. Testing for presence of a power law

After we concluded that our system possesses strong indicators of first-mover advantage, we next examine the presence of a power law, the key hallmark of self-organization. Specifically, we check if the distribution of total public spending per company can be described by a power law. As sketched in figure 7 in §3, for this task we use the approach proposed by Clauset *et al.* in [12].

We focus on several samples of companies: the entire set of 105 086 companies and four of its subsets (the biggest 20 000; biggest 15 000; biggest 10 000 and biggest 5000 companies in terms of total revenue). As the methods used in [12] are quite complex, we devote §4.2 to the details of it. It assumes that the examined distribution can be fitted with a power law in the form *x*^−*α*^ for a certain *α* > 0, and a certain minimal value *x*_min_ from which the fit is plausible. Hence, the first step is to estimate the values of *α* and *x*_min_. In table 1, we show these values for all samples of companies.

**Table 1.** The values *n* (number of companies), *x*_min_ and *α* for selected subsets of companies. Reported also are the corresponding *p*-values, whereby statistically significant values are denoted in italics.

sample	*n*	*x*_min_	*α*	*p*-value
all companies	105 086	272 087.52	1.64	5 × 10^−5^
biggest 20 000	20 000	349 365.49	1.64	1 × 10^−6^
biggest 15 000	15 000	62 444 576.36	1.84	*0.60*
biggest 10 000	10 000	62 444 576.36	1.84	*0.60*
biggest 5000	5000	62 444 576.36	1.84	*0.60*

The second step in §4.2 is to compute the goodness-of-fit between the data and the hypothesized power law for *α* and *x*_min_. This fit comes with a *p*-value: if *p* > 0.1, a power law is a plausible fit, but otherwise it should be rejected. We obtained *p* = 0.60 for the biggest 15 000, 10 000 and 5000 companies, and almost zero for the whole set of companies and biggest 20 000 companies, as reported in the last column of table 1. We find that the power law is a reasonably good fit for the biggest 15 000, 10 000 and 5000 companies, but not for the biggest 20 000 and more. It seems that somewhere between the biggest 15 000 and 20 000 companies, the power law stops being a plausible fit.

Finally, the third step in §4.2 is to see if any other heavy-tailed distribution can fit our data better than the power law (we limit ourselves to lognormal, exponential and stretched-exponential distributions). To check whether a given alternative distribution fits the data better, we perform a likelihood ratio (LR) test. This yields its own *p*-value and LR, shown in table 2. A positive LR indicates plausibility of a power law fit, while a negative LR indicates plausibility of the alternative over the power law. Corresponding *p*-values have to be smaller than 0.1, so that LR is statistically significant for the judgement. Our final judgement is given in the last column of table 2. Here, *‘none’* means that the data are probably not power-law distributed, but alternatives might be possible, while *‘moderate’* means that a power law is a good fit, but there might also be alternatives that describe the data distribution well enough. For our case of the biggest 15 000 companies and less, a power-law distribution wins over all competitors. In contrast, for the biggest 20 000 and more, we find no support for the power law. Instead, the most plausible fit here is the lognormal distribution.

**Table 2.** Tests of power-law fit in several samples of companies (see text). For each, we report the corresponding *p*-value for the fit to the power law, as well as likelihood ratios (LR) and their corresponding *p*-values. Statistically significant *p*-values are denoted in italics.

	power law	lognormal		exponential		stretched exp.		support for power law
sample	*p*-value	LR	*p*-value	LR	*p*-value	LR	*p*-value	
all companies	5 × 10^−5^	−5.6	2 × 10^−8^	16.90	2 × 10^−64^	0.95	0.34	none
biggest 20 000	1 × 10^−6^	−5.01	5 × 10^−7^	16.30	1 × 10^−6^	0.73	0.46	none
biggest 15 000	*0.60*	−0.19	0.85	7.65	2 × 10^−14^	1.22	0.22	moderate
biggest 10 000	*0.60*	−0.19	0.85	7.65	2 × 10^−14^	1.22	0.22	moderate
biggest 5000	*0.60*	−0.19	0.85	7.65	2 × 10^−14^	1.22	0.22	moderate

**Figure 5.**
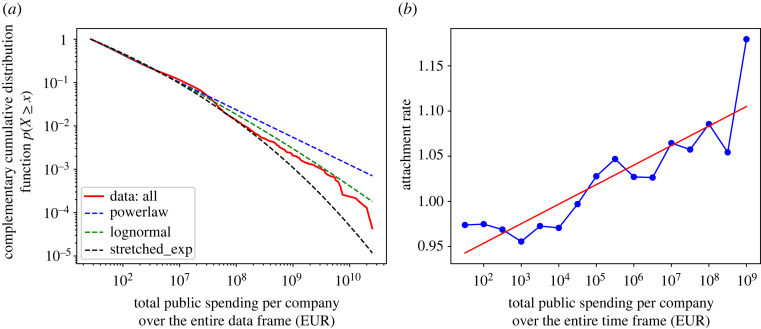
(*a*) Complementary cumulative distribution function (tail distribution) of our empirical data and the respective power-law, lognormal and stretched-exponential fits. (*b*) Average attachment rate *A*(*y*) versus total spending over the entire period on *x*-axis (log scale).

To zoom in better on the distribution for all companies, we show all three of the above-discussed fits in figure 5*a*. It is obvious that lognormal (green dashed line) best follows our empirical data. We calculated the parameters and obtained $f\;(x)=x^{0.096-1}\;e^{-284\;557\;x^{0.096}}$ for lognormal, and *f*(*x*) = *x*^−1.64^ for the best power-law parameter fit. We note that while lognormal distribution is not considered a strong indicator of self-organization, it still shows that the underlying process has certain regularities. Also, this figure suggests that a power law is still not a completely implausible fit. The plots for samples of the biggest 15 000 companies and less are available in the electronic supplementary material.

### 2.3. Testing for presence of preferential attachment

In our previous sections, we show that our public spending system is growing in a way that companies that came first had an obvious advantage over the rest and that there is plausibility for a power-law fit in the 15 000 top-ranked companies. This enables testing of the preferential attachment hypothesis [33] in our system, which leads us to the last question—*Q3*. We use Redner’s approach [41] to calculate the *attachment rate* *A*(*y*) as a function of total spending, as outlined in figure 7 and §4.3. Specifically, attachment rate is calculated as *A*(*y*) = Δ*y*/*y*, where *y* represents total spending per company in year *t*, and Δ*y* in the year *t* + 1, for *t* = 2003, 2004, …, 2018. Results are shown in figure 5*b*. The average is depicted over the entire period (blue line). The solid red line is the linear fit *P*(*x*) ∼ *a* + *bx*, with *a* = 0.91042, $b=0.02162,se(\hat{a})=0.018,se(\hat{b})=0.003$ and *R*^2^ = 0.78 (‘*se*’ stands for the standard error of the estimation). Therefore, we conclude that our system follows a linear preferential attachment mechanism, as evidenced by the linear relationship between attachment rate and total public spending per company. Together with the evidence for a power law and the results from previous sections, we can safely confirm at least some presence of self-organization in our system. This, however, does not mean that self-organization is the sole mechanism behind this process, nor that any divergences from it are necessarily proof against it. We consider this further in the Discussion.

### 2.4. Descriptive statistics and entry–exit analysis

In addition to our main findings, we provide more descriptive statistics on the temporal behaviour of our system. This approach affords readers a more holistic understanding of the data. Specifically, we look at how the companies behave in terms of entering and exiting our system over time.

**Figure 6.**
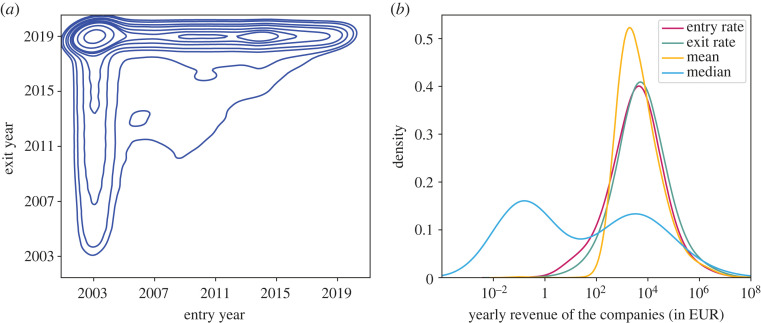
(*a*) Kernel density estimate (KDE) plot of entry versus exit year of all companies. The entry year is when the company received its first payment, while the exit year is when it received its last payment. Denser circles indicate that more companies share the same pattern, i.e. the same entry and exit years. (*b*) Probability density function of entry rates (the revenue the companies received in their entry years), exit rates (the revenue the companies received in their exit years), mean (average annual revenue per company) and median (of annual revenue per company).

Findings are shown in figure 6*a*, where it is evident that the majority of companies were active throughout the entire time span, from 2003 to 2019 (i.e. they joined the system in 2003 and stayed until the end of our dataset). The next largest group of companies entered the system between 2008 and 2015 and remained active until the end. On the other hand, there are companies that entered in 2003 but remained only for a few years. Finally, there are a few companies that both entered and exited sometime in between, spending only a few years in our system.

Figure 6*b* presents the distribution of entry and exit rates, in addition to the mean and median of yearly public revenue. The first three variables exhibit a similar pattern. This implies that most companies enter the system with an average revenue of 10k EUR per year, receive a comparable revenue each year while they stay in the system, and eventually exit the system receiving a similar revenue in the last year. From the median distribution, it is apparent that there are two primary peaks: one that indicates a large group of companies that received no payments at least half of the time, and a relatively smaller group that received around 10k EUR at least half of the time. These results indicate that most companies roughly stagnate over time, receiving a similar revenue from year to year. Meanwhile, a relatively small number of ‘key players’ seamlessly attract greater revenue.

## 3. Discussion

We studied publicly available data from the CPC of the Republic of Slovenia, with the goal of better understanding the driving mechanisms behind the country’s public-to-private spending.^4^ Hypothesizing self-organization as one of its leading mechanisms seemed rather unpromising in light of the ‘human nature’ involved in it. Nevertheless, our hypothesis, established along the lines of Schumpeter [5] and Gibrat [6], prevailed. In particular, we have shown that the distributions of total spending could be fitted reasonably well by a power law or a lognormal function. Moreover, the attachment rate of public spending to companies over time is roughly a linear function of total spending during the entire period. We have also shown evidence in favour of first-mover advantage [31,32]. As it turns out, the companies that started doing business with Slovenia between 2004 and 2007 are still those that, even after all these years, receive the most generous annual amounts of spending. Overall, this represents persuasive evidence in favour of self-organization in Slovenian public spending, as the title of our work suggests.

In a way, the above findings speak in favour of the fairness of Slovenian government bodies: they award larger business deals to companies that have already proven themselves by successfully completing similar sized deals. However, let us for a moment put a cynical hat on and entertain a disbelief in the integrity of political action in Slovenia. Are there alternative mechanisms that might give rise to the same statistics? One could reasonably expect that the leverage and lobbying power of any private company grows with its success in making business deals, with the government or otherwise. Various sectors, ranging from finance to the meat industry, are ripe with conflicts of interest, where previous incumbents assume powerful positions to ‘guide and advise’ on investment strategy and company policy. It would be naive not assume that this translates to proportional growth, but driven more by all-too-human motives and agendas rather than by pure self-organization. This would fit to our data too, given that older companies—and one has to assume that the very fact a company exists for a long time is because it is successful and growing—are those that attract the most funding. To give substance to such pondering, however, would require onymous data, with ample insight into the leading structures of companies and their political histories. To some degree, such data occasionally leaks online, is mentioned in impactful documentaries, or put together through careful and laborious gathering of various sources [42]. Unfortunately, more often than not, such information is not available, especially for scientific purposes.

Nevertheless, somewhat contrasting to this is our result that larger companies—the biggest 15 000 or less—seem to be more governed by self-organizing principles than smaller companies. This means that departures from perfect self-organization are more prominent for smaller companies, for whatever reason it may be. We believe this could be explained independently from the corruption-versus-integrity debate. Namely, none of the companies, large or small, are constrained to do business exclusively with the state. Instead, (probably) all of them are operating in the market too, and possibly do business with states other than Slovenia. None of this is reflected in our data, meaning that it may well be that smaller companies tend to do most of their business on the market and only occasionally interact with public budgets. If we also had at our disposal data on actual market spending, the results on self-organization might have been very different. But private-to-private spending is under no obligation to be reported to the CPC and hence is not at our disposal.

A separate methodological challenge is presented by the transactions that were not reported to the CPC. This behaviour is illegal in Slovenia, but unfortunately, we have no way of learning about this part of the relevant data. We estimate that such transactions do not represent a major fraction in the overall public-to-private spending. Still, we have no way of precisely measuring how large this fraction is, nor what its impact would be on the self-organization found in our system. An interesting speculation involves the idea that this might be (part of the) data missing for precise self-organization.

We finish with several ideas for future work. One of them is to cluster these companies (their time series) according to their business logic. This amounts to extracting commonalities in their dynamics and grouping them accordingly. This is the main avenue of our future work, which we may eventually extend using newer methods of time series analysis (e.g. shapelets [43]). On the other hand, the CPC also keeps track of specific business sectors behind each transaction in our dataset. Having this data would allow us to examine which businesses profit most from the state, how this changes from one government to the next, etc. Other ideas involve complementing our data with private-to-private transactions. Since tax must be paid for each such transaction, this data is available. We attempted to obtain it but were unsuccessful (so far). Finally, one may wonder how the state generates the funds it later spends on the private sector, and if these two processes are in any way related.

## 4. Methods

**Figure 7.**
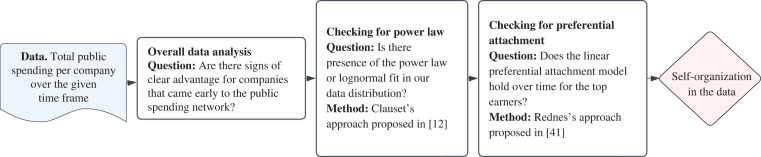
Chart flow of all the steps used for testing the presence of self-organization in public spending on private companies in Slovenia.

In this section, we provide more technical detail about our methodology used to obtain the results described in the previous section. We begin by schematizing the approach we used to argue in favour of self-organization. The steps we followed are shown in the form of a flow chart in figure 7. In the reminder of this section, we dive deeper into details of every method used and explain how our research framework can be reproduced.

### 4.1. Data and reproducibility

The dataset used for this study is available in our Dryad repository at https://doi.org/10.5061/dryad.5x69p8d6x. The complete code that support the findings is available in the same repository and is divided into three *.py* files. First-mover advantage analysis presented in §2.1 can be reproduced by running *first_mover.py* script from our Dryad repository. Programming codes for other parts of our methodology are also available, as described below.

### 4.2. Power law

To test if a certain variable is distributed according to the power law is more complicated than making a simple least-squares fit. There has been quite some debate about this, but luckily, the literature now offers several ways to test this in any system. We availed ourselves of the method proposed by Clauset *et al.* in [12]. For calculating all presented results in §2.2, we use *powerlaw: A Python Package for Analysis of Heavy-Tailed Distributions* developed by Alstott *et al.* [44], since it follows the approach of Clauset *et al.* [12]. Obtained results can be reproduced by running the *powerlaw_fit.py* script.

Mathematically, *x* obeys a power law if it is drawn from a probability distribution: *p*(*x*) ∼ *x*^−*α*^, where *α* is a constant parameter of the distribution known as the exponent or scaling parameter. According to the recipe from Clauset *et al.* [12], there are three main steps for analysing power-law fit.

#### 4.2.1. Estimating *x*_min_ and *α*

Most often, the power law applies only for values greater than some minimum *x*_min_, so the first step is to estimate *x*_min_ and the scaling parameter *α*. For this task, proposed methods are explained in section 3 of [12]. For *α* estimation, the proposed method is of *maximum likelihood*. For estimation of *x*_min_, we take the value that makes the probability distributions of the measured data and the best-fit power-law model as similar as possible above *x*_min_. As a measure for quantifying the distance between two probability distributions, the Kolmogorov–Smirnov statistic is used.

#### 4.2.2. Goodness-of-fit test

Given our dataset and a hypothesized power law from which the data are drawn, the next step is to check if a power law is a plausible fit for our dataset. For this task, proposed methods are explained in section 4 of [12]. We perform a *goodness-of-fit test*, which generates a *p*-value that quantifies the plausibility of the hypothesis. Such tests are based on measurement of the ‘distance’ between the distribution of the empirical data and the hypothesized model. As a measure of quantifying the distance, again the Kolmogorov–Smirnov test statistic [45,46] is used. Once we have our *p*-value, we need to make a decision about whether it is small enough to reject our null hypothesis, or to accept that the power law is a plausible fit. If the resulting *p*-value is greater than 0.1, the power law is a plausible hypothesis for the data, otherwise it is rejected. However, *p* close to 1 does not immediately mean that we have a power law; it only means that we have a case for plausibility of a power-law fit.

#### 4.2.3. Likelihood ratio test

This brings us to the final step: the LR test, whereby we compare our fit with other candidates. For consistency with [12], we selected these possible fits to be lognormal, exponential and stretched exponential (of course, one could have included more possibilities). Our goal here is to rule out these competitive distributions. To this end, we calculate logarithm values of the LRs between each two candidates: a power law and the alternative. Each such comparison comes with a corresponding *p*-value. The sign of LR indicates whether the alternative is more favoured than the power law: a positive LR means that the power-law fit is better, whereas a negative LR means that the alternative is better. As for the respective *p*-values, if *p* is small enough (*p* < 0.1), then we have a reliable indicator of which fit is better. By contrast, if *p* is large, then the sign of LR is not a reliable indicator of which fit is better.

### 4.3. Preferential attachment

Based on the assumption that the dynamics of our public spending system may be characterized by the mechanism where the company that received more in the given year will be more likely to receive even more in the following years, we have determined the average attachment rate following Redner’s approach described in [41] as follows. We calculated the attachment rate as *A*(*y*) = Δ*y*/*y*, so it gives the likelihood that a company with total revenue of *y* EUR in a given year will have Δ*y* more EUR in the next year. For even better understanding, we suggest looking at Figs. 1 and 2 in [41]. Our obtained results are shown in §2.3 and figure 5*b*, and they suggest that *A*(*y*) is a linear function of *y*. The results can be reproduced by running the *preferential_attachment.py* script from our Dryad repository.

## Data Availability

The data and the code that support the findings of this study are available in the Dryad Digital Repository: https://doi.org/10.5061/dryad.5x69p8d6x [47]. Supplementary material is available at [48].

## Footnotes

1 Public money in Slovenia is spent by a range of public institutions. They include national and regional governments, ministries, education institutions, hospitals, etc. See [49] for the entire list of such bodies.

2 A note on ‘all transfers/all transactions of public funds’: the CPC displays the transfers that it receives from the Republic of Slovenia Public Payments Administration. This means that transfers involving companies that are not fully state-owned are not included in the displayed data. Additionally, the CPC applies filters to the received transfers to comply with regulations protecting personal data, ensuring that only appropriate information is shown.

3 We can think of a violin plot as hybrid between a box plot and kernel density plot [50]. It shows peaks in the data and also depicts summary statistics and the density of each variable.

4 In 2022, the CPC updated the identification system for entities, switching from tax numbers to identification numbers, which now produces different results for the allocation of public funds to each entity. To address this, the CPC is currently enhancing the Erar application, scheduled for completion by the end of 2024. Consequently, it is important to note that the data available now may differ from what was observed and processed in this work.
